# Adherence to physical activity guidelines in mid-pregnancy does not reduce sedentary time: an observational study

**DOI:** 10.1186/s12966-015-0191-7

**Published:** 2015-02-24

**Authors:** Diana R Di Fabio, Courtney K Blomme, Katie M Smith, Gregory J Welk, Christina G Campbell

**Affiliations:** Department of Food Science and Human Nutrition, Iowa State University, 220 MacKay Hall, Ames, Iowa 50011 USA; Department of Kinesiology, Iowa State University, 235 Forker Building, Ames, Iowa 50011 USA; Interdepartmental Graduate Program in Nutritional Sciences, Iowa State University, Ames, Iowa 50011 USA; Sandy S. and Roy W. Uelner Professor of Food Science and Human Nutrition, Ames, Iowa 50011 USA

**Keywords:** Sleep, Activity patterns, Sedentary, activPAL™, Sensewear® Mini armband

## Abstract

**Background:**

Physical activity (PA) interventions designed to prevent prenatal complications have focused on increasing moderate PA yielding conflicting results. Minimal attention has focused on the evaluation of sleep, sedentary behavior (SB), light activity or total daily PA during pregnancy. The purpose of this prospective, longitudinal study was to 1) objectively quantify and compare habitual PA and SB during the 2^nd^ and 3^rd^ trimester; and 2) evaluate differences in activity patterns for women meeting prenatal PA guidelines versus those that did not.

**Methods:**

Forty-six participants wore 2 PA monitors (SenseWear® Mini and activPAL™) during week 18 and week 35 of pregnancy. We compared differences in sleep duration, postural allocation, daily steps, and PA between the 2^nd^ and 3^rd^ trimester and for women who met and did not meet PA guidelines.

**Results:**

During the 2^nd^ trimester, 30% of the women’s day (24-hours) was total sleep; 52% SB; 13% light; 3% moderate; and 0% vigorous PA. Light (P = 0.05), vigorous (P = 0.02), and moderate-vigorous PA (MET-minutes; P = 0.02), decreased with a trend in increased SB (P = 0.07). Activity of other intensities and sleep duration did not significantly change. Only 39% and 37% of participants slept between 7–9 hours/night at week 18 and 35, respectively. Forty-six percent (n = 21) and 28% (n = 13) of participants met prenatal PA guidelines during the 2^nd^ and 3^rd^ trimester, respectively. At week 18, no differences in total sleep, SB, or light PA existed for women who met PA guidelines versus those who did not; total PA was significantly greater for women who met guidelines. At week 35, women that met PA guidelines had significantly less SB (P < 0.005) than women who did not.

**Conclusions:**

This study demonstrates that pregnant women spend the majority of their day in SB. Significant reductions in total activity across pregnancy may be attributed, in part to shifts in light PA and increased SB. Based on the lifestyle of our sample, regardless of meeting PA guidelines in mid-pregnancy, no significant difference exists in time spent in SB, however meeting PA recommendations in late pregnancy may reduce SB. Future interventions should target reducing SB by increasing light and moderate PA beyond volitional exercise.

## Background

An infant’s risk of developing chronic disease later in life is influenced by the intrauterine environment established during pregnancy. Similarly, maternal health is an important predictor of an infant’s future risk of developing obesity [1]. Promoting preventative lifestyle strategies in prenatal care may be an effective way to curtail the rise in chronic disease. Physical activity (PA) during pregnancy has been identified as a potential approach to reduce the risk of prenatal complications such as excessive gestational weight gain (GWG) [2,3], abnormal glucose tolerance [4], gestational diabetes mellitus [4], pre-eclampsia [5], pre-term birth [6,7], and large- and small-for-gestational age infants [1,8], which increase the risk for future chronic disease. Although benefits of PA during pregnancy have been extensively documented, only about 25% of women in the United States [9] meet the 2008 Department of Health and Human Services prenatal PA guidelines of at least 150 minutes of moderate PA spread throughout the week [10]. Most PA interventions designed to prevent excessive prenatal weight gain have focused on increasing moderate PA [3,11-13]. However, minimal attention has been given to the evaluation of sleep, sedentary behavior, light activity or total volume of daily activity during pregnancy.

Non-exercise activity thermogenesis (NEAT) represents energy expended from behaviors as part of activities of daily living other than sleeping, eating, or volitional exercise [14]. NEAT, increases metabolic rate and thus, has been shown to be an important factor in the regulation of body weight in non-pregnant adults as a significant contributor to total daily energy expenditure [14]. Minimal attention has been given to the possible impact NEAT may have on perinatal outcomes despite recognition of future research needed in this area [15].

Increases in sedentary behavior (SB) during pregnancy have been associated with adverse perinatal health outcomes including abnormal glucose tolerance and increased risk for gestational diabetes mellitus [4], decreased insulin sensitivity and increased insulin secretion [16], excessive GWG [2], and lower birth weight [6]. Increasing time spent in light activity (i.e. NEAT) could have important health implications during pregnancy by directly reducing time spent in SB. Some studies have used self-report measures to quantify SB during pregnancy [4,6] but these may not have sufficient precision to distinguish SB from light PA [17]. Other studies have used objective devices but these have not separated nighttime sleep from total SB [2,18]. To advance work on the contributions of light or NEAT activity during pregnancy it is important to utilize objective methods that can distinguish sleep from total daily SB.

The present study advances research in this area by employing two state-of-the-art objective activity monitors for multiple, consecutive 24-hour periods to assess free-living PA and SB during pregnancy. The specific purpose of this prospective, longitudinal study was 1) to objectively quantify and compare habitual PA and SB during the 2^nd^ and 3^rd^ trimester; and 2) to evaluate differences in activity patterns for women meeting prenatal PA guidelines versus those that did not. The study will help to characterize patterns of SB and light PA during pregnancy to inform the best design of future prenatal interventions that aim to improve maternal and fetal outcomes.

## Methods

### Participants

Healthy pregnant women were recruited from local obstetric clinics, campus-wide emails, advertisements, and a partnership with a large hospital in a nearby city. Fifty-six women were enrolled in the prospective, longitudinal study at week 18 (±1 week) of gestation; 8 of these women did not complete the study for the following reasons: time constraints (n = 6), skin irritation from an activity monitor (n = 1), and pre-term delivery (n = 1). Inclusion criteria included 18–45 years of age and singleton pregnancy whereas the exclusion criteria included smoking during pregnancy or a history of chronic disease. Qualification criteria were confirmed by each participant’s medical provider. All participants provided written informed consent (approved by Iowa State University Institutional Review Board).

### Data collection

Data collection occurred for 7-consecutive days at week 18 (±1 week; 2^nd^ trimester) and week 35 (±1 week; 3^rd^ trimester) of gestation. No advice was provided during the study regarding prenatal exercise. At enrollment, height (Ayrton 226 Hite-Rite Precision Mechanical Stadiometer, quick Medical GS, Snoqualmie, WA) and weight without shoes or bulky clothing (Detecto Model 6855 Cardinal Scale, Manufacturing Co., Webb City, MO) were measured to the nearest 0.1 cm and 0.01 kg, respectively. Each participant was instructed to record PA in a 7-day record (PAR) and to wear 2 PA monitors (SenseWear® Mini armband (SWA), and activPAL™ for 7 days, 24 hours a day during each data collection period except when showering or swimming. To control for differences in the time of day participants began wearing the monitors, the data was standardized to represent 6, 24 hour periods and involved removal of data on the first and last day of the 7-day monitoring periods, as those provided partial days of data. Therefore we analyzed data from the 7-day period starting at midnight on the 1^st^ day of data collection and ending at midnight on the 6^th^ day of data collection for each participant.

### Activity monitors

#### SenseWear® Mini Activity Monitor (SWA)

The SWA (BodyMedia, Pittsburgh, PA) is a multi-sensor, pattern-recognition monitor that is worn on the left arm over the triceps muscle. It has unique potential for evaluation of pregnant women since it is worn on the arm, providing a more comfortable location than waist placement which has been shown to result in decreased compliance across pregnancy [19-21]. Good agreement between SWA estimates of energy expenditure and measured energy expenditure using an indirect calorimeter has been previously reported at mid-pregnancy using an earlier algorithm (version 5.2e; r = 0.93) [22]. These analyses have been repeated to show improved agreement and no systematic bias using the most currently available algorithm (version 5.2 h, unpublished observations from C. Campbell). Data were downloaded using version 8.0 of the BodyMedia software (algorithm v5.2 h). An excel code was written to categorize minute epochs into sleep, sedentary (≤1.5 METs; independent of nighttime sleep), light (1.6-2.9 METs), moderate (3-5.9 METs), vigorous (≥6 METs) PA, and PA volume (total MET-minutes per day and daily moderate-vigorous PA (MVPA) MET-minutes) [23,24].

**Figure 1:**
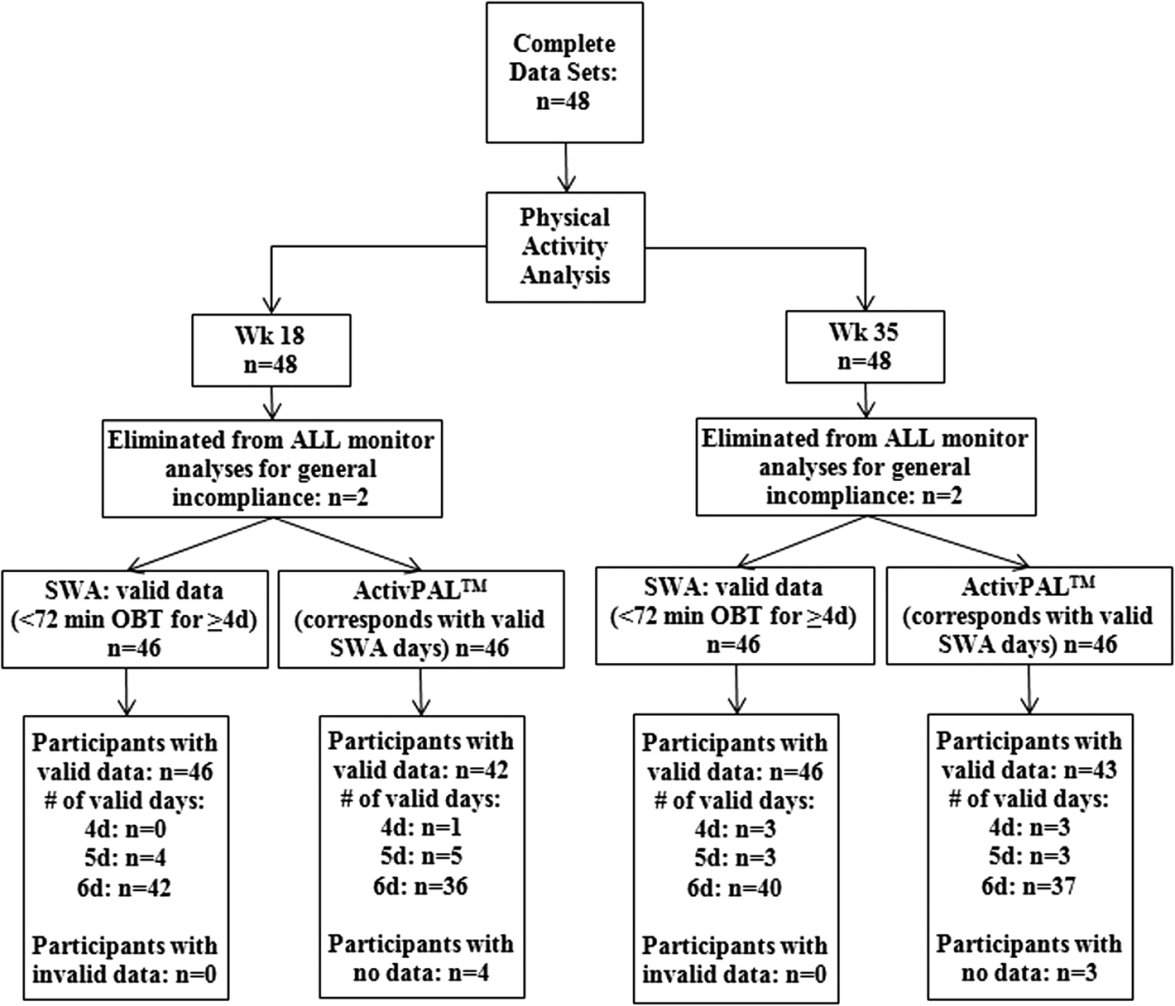
**Number of participants included in the analysis for each physical activity monitor.** SWA, SenseWear® Mini Armband. Physical activity data was evaluated at each time point using criteria for valid data to determine inclusion in the analysis of SWA or activPAL™ files.

An advantage of the SWA is that the monitor automatically detects when the monitor is not worn, also known as off-body time (OBT; e.g. due to showering or swimming). A “valid” day was defined as less than 72 minutes of OBT and at least four valid days were required [25]. OBT in excess of 72 minutes was evaluated using the PARs (Figure 1). If OBT included water exercise (e.g. water aerobics: 5.5 MET, code 18355), a MET was assigned from the 2011 Compendium of Physical Activities [24] and time spent in these activities supplemented the objective PA data (n = 10 participants). The SWA has been shown to reliably quantify sleep when compared to polysomnography [26], the gold standard for objective sleep measurement. Nighttime sleep was identified from the SWA and defined as sleep between 10 pm and 7 am. Sleep extending beyond the timeframe of 10 pm – 7 am was only counted as nighttime sleep if sleep was uninterrupted for more than two hours (e.g. sleep from 11 pm-6 am, and 7-8 am was counted as 8 hours of nighttime sleep). None of the participants worked overnight shifts that would result in abnormal sleep patterns.

Data from the SWA was used to assess adherence to PA recommendations. This was done in two ways to account for various interpretations of the 2008 Department of Health and Human Services prenatal PA recommendations: 1) ≥ 150 minutes of accumulated MVPA per week and 2) ≥ 150 minutes of MVPA per week completed in at least 10-minute bouts [22]. A 10-minute bout consisted of at least 8 moderate-vigorous minutes within 10-consecutive minutes thereby allowing for up to 2 minutes below the moderate intensity threshold as previously reported [18]. Vigorous activity was counted as two minutes of moderate PA [10].

#### activPAL™

The activPAL™ (PAL Technologies, Ltd, Glasgow, Scotland) is an innovative accelerometer designed to evaluate postural allocation [27], offering considerable potential for understanding SB. Unlike the SWA, a key feature of the activPAL™ is the ability to differentiate time spent lying down/sitting versus standing or walking. However, sedentary time reported by the activPAL™ includes sleep (nighttime sleep plus naps) as this monitor is not able to discern between sleep and wake time spent sitting or lying. As such, data from the activPAL™ are presented as sit/lie and upright (standing and stepping). The term sedentary was not used because sleep is included in sit/lie time and is a biologically necessary sedentary behavior. To summarize the differences between these two monitors, the activPAL™ identifies SB based on posture alone (e.g. sit versus stand), whereas the SWA defines SB according to METs (e.g. ≤ 1.5 METs).

The activPAL™ is worn on the right leg over the quadriceps muscle with an adhesive provided by the manufacturer. This monitor has been used successfully in various populations [27-31] and has been validated to quantify postural allocation and step counts [27]. activPAL™ data were analyzed according to previously published methodology [28] and variables of interest included daily totals of steps, sit/lie, upright, standing, and stepping time, and number and length of sit/lie and upright bouts (Note: a bout is operationalized as any period of time greater than one second during which a posture was maintained). The same days used to assess SWA data were used to analyze the activPAL™ data. Three participants with valid SWA data did not have activPAL™ data; these participants’ SWA data was retained in the analysis. Additionally, two women had inadequate SWA wear time (see *SenseWear® Activity Monitor*) and were excluded from analyses at both time points for both monitors; activPAL™ cannot distinguish non-wear time from sit/lie time therefore it was assumed if the SWA was not worn, the activPAL™ was also not worn. Thus, 46 complete data sets were assessed for the SWA (Figure 1). Since six participants did not have any activPAL™ data at week 18 and/or week 35, 40 complete data sets were assessed for the activPAL™ (Figure 1).

### Data analyses

Descriptive statistics were used to assess participant characteristics. The Shapiro-Wilk test for normality revealed the majority of the data from the SWA was normally distributed while the data from the activPAL™ was not; therefore values were reported in means and standard deviations or medians and interquartile ranges (IQRs), respectively. PA and SB variables across pregnancy were compared using either paired t-tests (SWA) or Wilcoxon rank-sum test (activPAL™). Independent t-tests with a Bonferroni adjustment for multiple comparisons were used to analyze any differences in PA variables between participants meeting PA guidelines versus those that did not. Significance was set at P < 0.05 and analyses were conducted with NCSS 2007 (Number Cruncher Statistical System; version 07.1.20, NCSS, LLC., Kaysville, Utah).

## Results

### Participant characteristics

Participants were young adults (mean age = 29.0 ± 3.5 years old), predominantly married (93%) and Caucasian (93%). All had some college education, and 54% were nulliparous. Overall, participants had an average pre-pregnancy body mass index (BMI) of 24.9 ± 5.0 kg/m^2^ (underweight (<18.5 kg/m^2^) BMI: n = 1; normal (18.5-24.9 kg/m^2^): n = 30; overweight (25–29.9 kg/m^2^): n = 9; obese (>30 kg/m^2^): n = 6).

### Physical activity and sedentary behavior patterns

**Figure 2:**
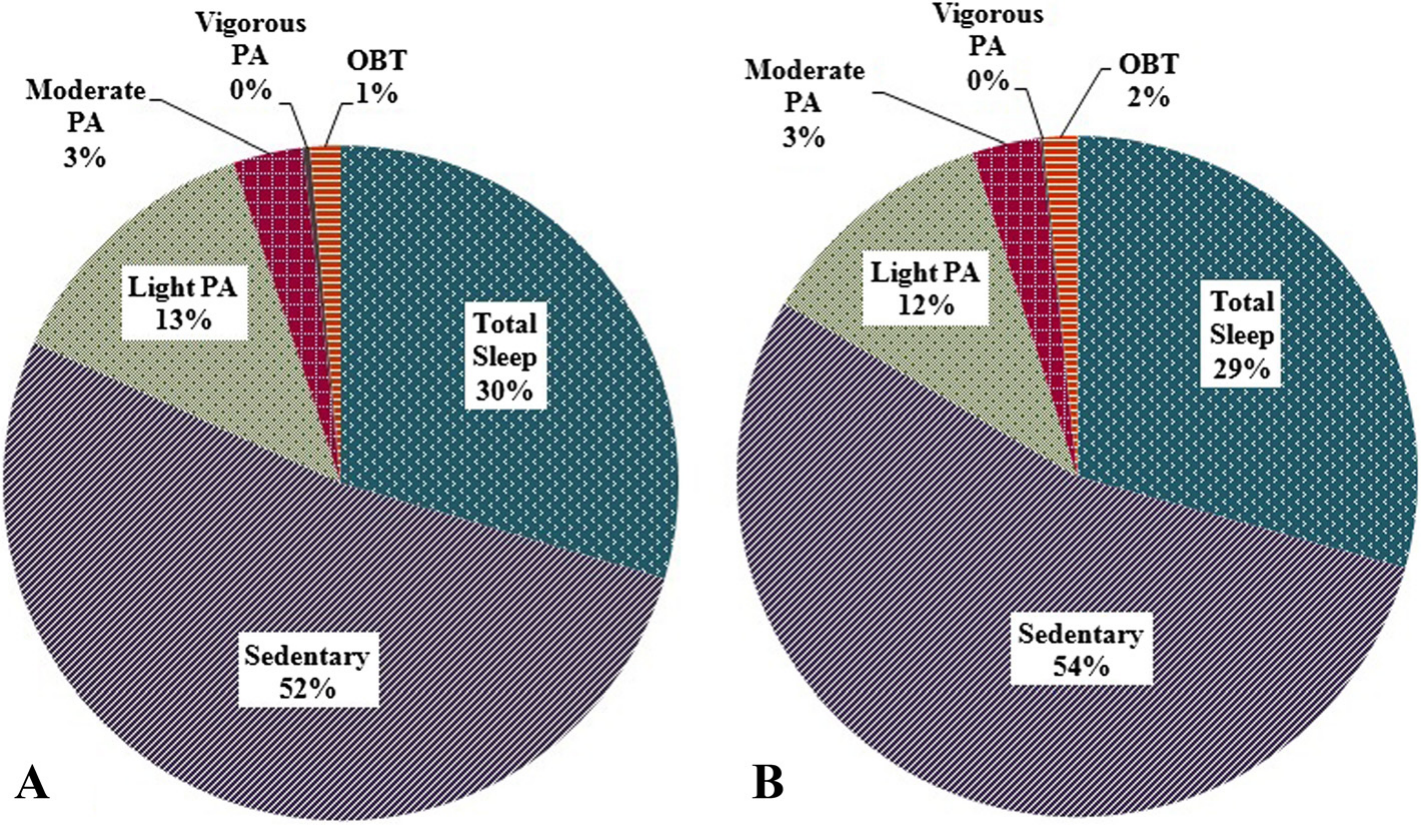
**Daily profile of activity per the SWA during A) 2** ^**nd**^ **trimester and B) 3** ^**rd**^ **trimester.** PA, physical activity; OBT, off-body time.

According to the SWA, the percentages of a day spent in SB, light PA, moderate PA, vigorous PA, and total sleep, at week 18 and 35 are depicted in Figure 2A and B, respectively. Light and vigorous PA significantly decreased from week 18 to week 35 (P = 0.05 and P = 0.02, respectively) while naps, accumulated moderate PA, and MVPA in bouts of at least 10 minutes did not significantly change between time points. For all participants combined, there was a trend for sedentary time (including napping) to increase from week 18 to week 35 (P = 0.07). Total MET-minutes per day and MVPA MET-minutes in at least 10-minute bouts significantly decreased from week 18 to 35 (Table 1).

**Table 1:** **Sedentary behavior and physical activity during the 2** ^**nd**^ **and 3** ^**rd**^ **trimester (n = 46)**

**SenseWear® armband**	**Gestation length (weeks)**		**P-value** ^**a**^
	**Week 18**	**Week 35**	
Nighttime sleep (hrs∙d^−1^)	6.8 ± 0.8	6.7 ± 0.7	0.51
Naps (min∙d^−1^)	13 ± 15	14 ± 14	0.66
Total sleep (hrs∙d^−1^)	7.0 ± 0.9	7.0 ± 0.7	0.6
Sedentary (excludes all sleep) (hrs∙d^−1^)	12.4 ± 1.7	12.9 ± 2.2	0.07
Time awake in sedentary behavior (%)	76 ± 11	78 ± 13	0.05
Light PA (hrs∙d^−1^)	3.1 ± 1.4	2.7 ± 1.5	0.05
Accumulated moderate PA (min∙d^−1^)	48 ± 42	46 ± 57	0.75
Accumulated vigorous PA (min∙d^−1^)	5 ± 10	2 ± 4	0.02
Moderate-vigorous PA in ≥ 10 minute bouts (min∙d^−1^)	32 ± 41	27 ± 43	0.1
Moderate-vigorous PA MET-minutes in ≥ 10 minute bouts (min∙d^−1^)	150 ± 197	113 ± 179	0.02
Total MET-minutes (∙d^−1^)	1841 ± 266	1747 ± 283	<0.001

PA, physical activity; MET, metabolic equivalent of task. Significance was set at P < 0.05. Values reported as mean ± standard deviation. ^a^Paired t-test.

Thirty-nine percent (n = 18) of participants at week 18 and 37% (n = 17) at week 35 averaged between the recommended 7–9 hours of sleep per night [32]. Time spent sleeping at night did not significantly change (Table 1). None of the participants slept less than an average of 5 hours per night or greater than 9 hours at either time point yet 62% of women slept less than 7 hours per night at both time points.

Utilizing the activPAL™, no significant differences in sit/lie and upright time were observed between week 18 and 35; however, total stepping time, total steps per day, and the length of sit/lie and upright bouts all significantly decreased (P < 0.001, P < 0.001, P = 0.003, P < 0.001, respectively) (Table 2). In addition, the number of sit/lie and upright bouts per day, and the number of sit to stand transitions significantly increased across pregnancy (P < 0.005 for all variables).

**Table 2:** **Daily activity profile including sit/lie and upright time during the 2** ^**nd**^ **and 3** ^**rd**^ **trimester (n = 40)**

**activPAL™**	**Gestation length (weeks)**		**P-value** ^**a**^
	**Week 18**	**Week 35**	
**Sit/lie time**			
Sit/lie time (includes all sleep) (hrs∙d^−1^)	18.2 (17.1-19.0)	18.3 (17.6-19.4)	0.29
Sit/lie (% of day)	76 (71–79)	76 (73–81)	0.29
Number of sit/lie bouts (number∙d^−1^)	35 (25–44)	46 (30–59)	0.003
Length of sit/lie bout (min∙d^−1^)	32 (24–42)	22 (18–37)	0.003
Number of transitions between sedentary (sit/lay) to upright (∙d^−1^)	35 (25–45)	48 (31–65)	0.002
**Upright**			
Upright time (includes stepping & standing time) (hrs∙d^−1^)	5.8 (5.0-6.9)	5.7 (4.6-6.4)	0.29
Stepping time (hrs∙d^−1^)	3.5 (2.4-4.5)	2.1 (1.4-3.4)	<0.001
Standing time (hrs∙d^−1^)	2.1 (1.6-2.9)	3.3 (1.9-4.6)	0.001
Upright (% of day)	24 (21–29)	24 (19–27)	0.29
Number of upright bouts (∙d^−1^)	34 (25–45)	44 (30–59)	0.003
Length of upright bout (min∙d^−1^)	10 (7–14)	7 (6–12)	<0.001
**Steps**			
Steps (number∙d^−1^) ^,^	10,102 (7329–12,408)	7323 (6187–10,151)	<0.001

Significance was set at P < 0.05. Values reported as median (interquartile range).

^a^Wilcoxon rank-sum test.

### Adherence to physical activity guidelines

Using the definition of ≥ 150 minutes of accumulated MVPA, 65% and 61% of women met the guideline at week 18 and 35, respectively. With the definition of ≥ 150 minutes of MVPA in a bout of at least 10 minutes, 46% and 28% of women met the guideline at week 18 and 35, respectively. Those who met the guideline at week 18 spent an average of 344 ± 279 minutes in at least 10-minute bouts per week. After correcting for multiple comparisons, no differences in time spent in SB, light PA, or total sleep at week 18 were identified for women who met PA guidelines versus those that did not (Table 3). At week 35, no differences for total sleep and light PA persisted, yet those women that met PA guidelines had significantly less SB. However, those who met guidelines during either the second or third trimester spent about 70% of their time awake in sedentary behavior compared to 80% for women that did not meet guidelines. Similarly, women who met guidelines at week 18 and 35 had significantly greater daily MET-minutes (P < 0.001).

**Table 3:** **Women who met versus did not meet physical activity guidelines** ^**a**^ **during 2** ^**nd**^ **and 3** ^**rd**^ **trimester**

**SenseWear® armband**	**Met PA guidelines** ^**a**^	**Did not meet PA guidelines** ^**a**^	**P-value** ^**b**^
**Week 18**	**n = 21**	**n = 25**	
Total sleep (includes nighttime sleep and naps) (hrs∙d^−1^)	7.0 ± 0.7	7.1 ± 1.0	0.82
Sedentary (hrs∙d^−1^)	11.8 ± 1.7	12.9 ± 1.5	0.03
Light PA (hrs∙d^−1^)	3.3 ± 1.3	3.0 ± 1.4	0.46
Total MET minutes (∙d^−1^)	1981 ± 282	1724 ± 187	<0.001
**Week 35**	**n = 13**	**n = 33**	
Total sleep (includes nighttime sleep and naps) (hrs∙d^−1^)	6.8 ± 0.7	7.0 ± 0.7	0.46
Sedentary (hrs∙d^−1^)	11.5 ± 2.2	13.4 ± 1.9	0.005
Light PA (hrs∙d^−1^)	3.3 ± 1.1	2.5 ± 1.5	0.09
Total MET-minutes (∙d^−1^)	1999 ± 314	1647 ± 199	<0.001

Significance was set at P < 0.0125. Values reported as mean ± standard deviation; PA: physical activity.

^a^2008 Department of Health and Human Services prenatal physical activity guidelines; ^b^Independent t-tests with Bonferroni adjustment for multiple comparisons.

## Discussion

The current study demonstrates that the sampled group of healthy women with a low-risk pregnancy spent more than half their total day and at least 70% of their time awake in sedentary behaviors regardless of meeting current 2008 Department of Health and Human Services prenatal PA guidelines. In comparison, the general adult population has been reported to spend about 55-60% of time awake in sedentary behavior [29,30]. Contrary to previous findings of activity patterns during pregnancy [9,20,33], moderate PA did not change over time, however vigorous and total PA volume, represented by total MET-minutes and steps declined. Nighttime sleep remained inadequate with over 60% of the women sleeping less than seven hours per night.

Although research has targeted prenatal MVPA and volitional exercise as a means to minimize adverse prenatal outcomes, little attention has been given to behaviors during the rest of the day. Pregnant women placed on activity restriction (i.e. bed rest) represent a highly sedentary population. Bed rest is associated with maternal muscle atrophy [34], weight gain [34,35], bone loss [35], and low birth weight [36]. Thus, the considerable time spent in SB as demonstrated in the current study may be of great concern - reinforcing previous studies [2,4,6,16] relating increased SB to adverse pregnancy outcomes. Collectively, time spent in SB may be a crucial component of daily behavior that should be targeted in future interventions.

Both total PA and moderate PA have been show to decrease during the 3^rd^ trimester [9,33,37,38]. A previous report demonstrates a decrease in moderate PA across pregnancy via objective monitoring (ActiGraph accelerometer, model #AM7164) [33] while the current study demonstrates the change in total PA is possibly due to a reduction in light and vigorous PA rather than a decrease in moderate PA. One distinct difference between the opposing findings is the amount of time participants wore the activity monitors. In the current study, the participants wore the monitors 24-hours a day (except when submerged in water) yielding an average wear time of 23.6 hours per day compared to 12.3 hours per day in the comparative study [33]. If our sample is representative of pregnant women’s typical sleep patterns, approximately 17 hours of awake time (24 total hours – 7 sleep hours = 17 awake hours) exists each day outside of nighttime sleep, leaving over 5 hours a day not accounted for in partial-day monitoring. To best understand total PA, including SB, objective monitoring of the majority of day- and nighttime activities is imperative.

Two useful indicators of total PA are MET-minutes per day and steps per day which reflect the total volume of daily activity encompassing sedentary, light, moderate and vigorous PA. Higher levels of total daily activity have been associated with the prevention of excessive GWG such that women with > 8.5 MET-hours per week of activity of all intensities were less likely to gain excessive weight [39]. The current study provides support for future efforts to prevent prenatal complications by decreasing SB through increasing overall activity (emphasizing a reduction in SB, an increase of light PA or NEAT in addition to volitional exercise). A previous report from Gradmark et al. supports this idea since total activity, rather than subcomponents of PA, were determined to be most strongly associated with insulin sensitivity during pregnancy [16].

The 24-hour monitoring period was particularly valuable to capture and account for nighttime sleep, independent of daytime SB. Nighttime sleep should be assessed when considering optimal behaviors during pregnancy as disrupted sleep patterns have been reported to start in the first trimester and continue throughout pregnancy [40,41]. Sleep has been reported to be of poor quality, decreased duration, decreased efficiency, and more fragmented towards the end of pregnancy [40]. Borodulin et al. used a measure of self-report to determine that 61.3% of women (n = 1259) during their 2^nd^ trimester were sleeping between 7–9 hours per night, the recommended amount for adults [42]. Comparatively, in the current study using an objective assessment of sleep only 39% and 37% of participants met these recommendations during the 2^nd^ and 3^rd^ trimester, respectively. Sleep deprivation could lead to daytime napping, which may not fully compensate for inadequate nighttime sleep [43]. Additionally, regular daytime sleep could increase the risk for still birth [43] and maternal hyperglycemia [44]. Given the prenatal health concerns associated with inadequate sleep, it is important to assess behavior over a 24-hour period so that sleep and daytime PA can be evaluated in relation to health outcomes.

Sedentary time described by the activPAL™ includes all time spent lying or sitting, including sleep, whereas the SWA defines sedentary time based on energy expenditure. Therefore, these definitions explain the observed differences in sedentary time between the SWA and activPAL™. Occupation was not assessed and could influence changes in activity patterns across pregnancy. For example, early in pregnancy, an elementary school teacher may stand for longer bouts whereas later in pregnancy, she may opt to sit while teaching. Future studies could evaluate occupation as a possible covariate to explain changes in PA across pregnancy. Finally, it is important to note that the sample of pregnant women in the current study was small, highly educated, mostly Caucasian and married. Thus, the sample may not be representative of populations with more diversity in race and socioeconomic status.

## Conclusions

This study demonstrates that after accounting for total sleep, pregnant women spend more than half of the 24 hour day, or at least 70% of time awake, in sedentary behaviors. Additionally, women meeting prenatal PA guidelines did not have significantly less SB at mid-pregnancy. Throughout pregnancy, SB remained the most predominant daily behavior; while vigorous PA, steps, and MET-minutes significantly decreased in the 3^rd^ trimester. Significant reductions in total activity across pregnancy may be attributed in part to shifts in decreased light PA, increased SB, and a reduction in vigorous PA. Attention to maintaining an active lifestyle during pregnancy has focused on increasing MVPA; however, promotion of reducing sedentary time and increasing light and moderate activity beyond volitional exercise may be additional strategies to target in future interventions to promote optimal maternal and fetal health outcomes.

## Abbreviations

AP: ActivPAL™
GWG: Gestational weight gain
MET: Metabolic equivalents of task
MVPA: Moderate-vigorous physical activity
NEAT: Non-exercise activity thermogenesis
PA: Physical activity
PAR: Physical activity record
SB: Sedentary behavior
SWA: SenseWear® Mini armband
